# Annotations

**Published:** 1887-07-09

**Authors:** 


### Annotations.

**Wooden Hospitals.** The City of Aberdeen is rearranging almost the whole of its hospital accommodation. Surgeon-General Fasson has been called in as an expert to examine, report upon, and advise the management. Most people are aware that not only all the principal buildings, but the leading streets of Aberdeen, are built of granite, which is more abundant in the neighbourhood than any other kind of stone. Surgeon-General Fasson recommends that the new portion of the fever hospital be also built of granite. There are, however, some objections to this course on the ground of expense, in spite of the abundance of that stone in the immediate neighbourhood. As an alternative it is advised that wood be the material employed. A double case of wood, with an intermediate space filled with sawdust, it is said will answer all practical purposes. The only objection to this seems to be the increased risk from fire; but it is pointed out that this risk is comparatively small, other wooden buildings used for this and similar purposes not being apparently more liable to take fire than structures of stone or brick. It is worthy of note that provision is proposed to be made for paying patients: a step in the right direction, when it is considered how few are the conveniences, even in fairly good houses, for the nursing and management of prolonged cases of fever. The whole subject of the medical management of those suffering from fever is under general consideration, and it is highly probable that some more intelligent means of dealing with these cases will soon become general. Of course medical men who are already hard-pressed by the competition of the times will naturally object to the loss of work and emolument which seem to be here implied. But the proper course to pursue is so to arrange that the family medical man shall in every case attend the fever patient where it is so desired. If the business be intelligently managed there ought to be the most cordial agreement between hospitals and family practitioners.

**Convalescent Aid.** The care and management of the sick poor are daily becoming more thorough, efficient, and complete. *The Times* and other papers are opening their columns for the propagation of ideas and information relative to convalescents. The outside world has the idea that when a sick person is once admitted within the walls of a hospital he will either remain there until he is completely cured and fit for work, or until he dies or is pronounced incurable. This is by no means the case, as all the medical world very well knows. Such is the pressure upon the resources of the hospital, and such are the limits of those resources, that every case, with some exceptions, is dismissed from its care at the earliest possible moment. Those that are curable are restored to a certain degree of health as rapidly as possible, and as soon as they can be removed with safety are sent away. But most of the in-patients of hospitals belong to the working-classes, and in many cases, although their diseases are cured, they are several weeks distant from the time at which they should properly return to work. They often do work in this condition, with the result that relapses among them are frequent, and instead of rapid convalescence and perfect recovery, there is prolonged weakness and disability. Convalescent homes for this class of patients are very urgently needed, and it is true economy to provide them and make them easily available. We are glad to see that hospital managers and the charitable public are opening their eyes to these facts, and we may hope that at no distant time there will be for every enfeebled convalescent a period of repose and building up, which will enable him to return to his work with adequate vigour, and to continue it without break-down.

**A Krupp Gun Wanted.** A good many things are done in London for which it would be difficult to plead justification. If they merely concerned the doers of them it would be a matter of no concern to the public. There seems to be a passion for the building of hospitals, not only in neighbourhoods where they are needed, but in districts where no stretch of imagination can declare them to be necessary. These manifold ventures, started often in the interests and to gratify the ambition of aspiring medical men, receive more or less public support, thereby diminishing that given to established institutions. It is difficult to know how to deal with such cases. No Government action can be asked for in order to put a check upon them, and if it were it would not be granted. As a matter of fact there is no law, nor ought there to be any, to prevent people doing what they please in this way, even though every street in the city and every house in every street should be turned to hospital uses. Nevertheless, it is a very great misfortune, and causes serious injury to those hospitals which are already in existence and doing useful work. It may be stated generally that no man is justified in giving his name or help to any new hospital scheme unless it can be shown clearly that such a scheme is necessary for the well-being of the district where the hospital is proposed to be placed. For the most part these new ventures are initiated at the West-end, where hospitals of all kinds are as thick as blackberries. It is not that all the patients are crowded together there, or that the West-end is more easy of access than other parts of London, but that there ambitious specialists and beardless consultants live by the hundred, and are bent upon doing something to bring themselves into notoriety. We submit that hospitals are not intended for the convenience and advantage of doctors, but for the treatment and cure of the suffering poor.

